# Supplementary material for: Transcriptomic Insights into Mechanisms of Early Seed Maturation in the Garden Pea (Pisum sativum L.)
Source: Cells. 2020 Mar 23;9(3):779. doi: 10.3390/cells9030779 (PMC7140803; doi:10.3390/cells9030779)
Supplement: Supplementary file 1 [file cells-09-00779-s001.zip › Supplementary materials/FileS3.zip!/FileS2/Hormones - Results/Summary Sheets - Groups/0verview.html]

Group Summary of Hormones dataset


# Group Centered Analyses

The SOM portraits are aggregated in groupwise fashions. Further,
group stability scores are calculated using correlation silhouette method.
Finally, samples within each group are clustered and visualized as
hierarchical dendrograms.

- Expression Portraits (PDF)
- Assignment Stability Scores (PDF)
- Groupwise Clustering Dendrograms (PDF)

## Group Summary Sheets

For each group a report sheet, gene lists and gene set lists are
created analogous to the sample summary sheets. Additionally, specific
gene sets are given which show highest significance in statistical
testing of the group compared to all other groups.

| Group | Summary Sheet | Global Gene List | Gene Set List |
| --- | --- | --- | --- |
| Sprint-2(10) | PDF | CSV | CSV |
| Sprint-2(20) | PDF | CSV | CSV |
| Zhewan-1(10) | PDF | CSV | CSV |
| Zhewan-1(25) | PDF | CSV | CSV |
| Zhongwan-6(10) | PDF | CSV | CSV |
| Zhongwan-6(25) | PDF | CSV | CSV |
